# Bmi1 Loss in the Organ of Corti Results in p16^ink4a^ Upregulation and Reduced Cell Proliferation of Otic Progenitors *In Vitro*

**DOI:** 10.1371/journal.pone.0164579

**Published:** 2016-10-18

**Authors:** Mohamed Bassiouni, Aurélie Dos Santos, Hasan X. Avci, Hubert Löwenheim, Marcus Müller

**Affiliations:** Department of Otolaryngology, Head and Neck Surgery, Tübingen Hearing Research Centre, Eberhard Karls University Tübingen, Tübingen, Germany; Texas A&M University, UNITED STATES

## Abstract

The mature mammalian organ of Corti does not regenerate spontaneously after injury, mainly due to the absence of cell proliferation and the depletion of otic progenitors with age. The polycomb gene B lymphoma Mo-MLV insertion region 1 homolog (Bmi1) promotes proliferation and cell cycle progression in several stem cell populations. The cell cycle inhibitor p16^ink4a^ has been previously identified as a downstream target of Bmi1. In this study, we show that Bmi1 is expressed in the developing inner ear. In the organ of Corti, Bmi1 expression is temporally regulated during embryonic and postnatal development. In contrast, p16^ink4a^ expression is not detectable during the same period. Bmi1-deficient mice were used to investigate the role of Bmi1 in cochlear development and otosphere generation. In the absence of Bmi1, the postnatal organ of Corti displayed normal morphology at least until the end of the first postnatal week, suggesting that Bmi1 is not required for the embryonic or early postnatal development of the organ of Corti. However, Bmi1 loss resulted in the reduced sphere-forming capacity of the organ of Corti, accompanied by the decreased cell proliferation of otic progenitors in otosphere cultures. This reduced proliferative capacity was associated with the upregulation of p16^ink4a^ *in vitro*. Viral vector-mediated overexpression of p16^ink4a^ in wildtype otosphere cultures significantly reduced the number of generated otospheres *in vitro*. The findings strongly suggest a role for Bmi1 as a promoter of cell proliferation in otic progenitor cells, potentially through the repression of p16^ink4a^.

## Introduction

In the mammalian organ of Corti (OC), auditory sensory hair cells do not regenerate after injury, unlike in the auditory sensory epithelia of other vertebrates [1]. A primary underlying mechanism of this regenerative failure is the lack of cell proliferation in response to damage [2, 3]. However, when cells of the OC are dissociated and cultured, they proliferate and generate multipotent otospheres [4]. It is important to note that OC cells are normally quiescent *in vivo* [5] but are able to re-enter the cell cycle after dissociation and culturing. This behavior suggests that OC cells possess an intrinsic proliferative potential that is inhibited under *in situ* conditions. Thus, the identification of factors that regulate the cell cycle exit *in situ*, and cell cycle re-entry *in vitro*, may provide cues for the induction of hair cell regeneration.

The protein B lymphoma Mo-MLV insertion region 1 homolog (Bmi1) is a member of the polycomb group of proteins, which form repressive complexes that suppress gene expression through chromatin modulation [6]. Previous studies have shown that Bmi1 maintains the proliferation and cell cycle progression of several stem cell populations (reviewed in [7]), such as neural stem cells [8] and hematopoietic stem cells [9]. Bmi1-deficient mice suffer from neurological and hematopoietic deficits, presumably due to defects in neural and hematopoietic stem cells [10]. It was previously shown that Bmi1 promotes cell proliferation in several cell types through the repression of the ink4a/arf locus, which encodes two known cell cycle inhibitors, namely, p16^ink4a^ and p19^arf^ [11, 12]. Interestingly, p16^ink4a^ and p19^arf^ exert their anti-proliferative functions through distinct mechanisms. The tumor suppressor p16^ink4a^ inhibits the cyclin D-dependent kinases CDK4 and CDK6 [13]. In the absence of CDK4/CDK6-cyclin D complexes, the retinoblastoma proteins (Rb) remain in their inactive hypophosphorylated state [13]. Hypophosphorylated Rb, in turn, bind to elongation factor 2 (E2F) transcription factors, hindering their capacity to activate genes that are required for entry into the S-phase of the cell cycle [14]. The other product of the ink4a/arf locus, p19^arf^, inhibits proliferation by blocking the degradation of the tumor suppressor protein p53 [15]. In the present study, we focused on p16^ink4a^ as a potential downstream target of Bmi1 due to increasing evidence that implicates p16^ink4a^ in the age-related decline in the proliferative capacity of the brain [16], hematopoietic stem cells [17], pancreatic islets [18] and skeletal muscle [19].

Bmi1 expression has been shown in the embryonic cochlea [20] and the postnatal and mature OC [21]. Recent studies have reported that, although the neonatal Bmi1-deficient OC showed normal morphological development, Bmi1 loss resulted in a diminished sphere-forming capacity of the OC [21, 22]. However, it is unknown whether this reduced sphere-forming capacity is associated with ink4a/arf gene expression changes in Bmi1-deficient spheres. More importantly, it remains elusive why Bmi1 loss influences cell proliferation in the OC-derived spheres *in vitro*, in a manner that is not observed in the developing cochlea *in vivo*.

To investigate the function of Bmi1 in the OC, we utilized the homozygous Bmi1^GFP/GFP^ reporter mouse [23], which has been used as a Bmi1 loss-of-function model in previous studies [24, 25]. The otosphere assay was used as a tool to investigate the proliferative potential of the Bmi1-deficient OC. Our findings indicate that Bmi1 maintains the proliferative capacity of otic progenitors *in vitro* in association with p16^ink4a^ repression.

## Materials and Methods

### Animals and genotyping

Animal experiments were approved by the Tübingen Regional Council (Regierungspräsidium) (animal experiment approval HN4/14 and approval of animal use for organ explantation dated June 27, 2012 and July 27, 2015). All animals received care in compliance with the Directive 2010/63/EU on the protection of animals used for scientific purposes.

All of the animals were housed in an in-house animal facility at the University of Tübingen. C57Bl/6 mice were purchased from Charles River Laboratories (Sulzfeld, Germany) (Jax stock number 005304). Bmi1-GFP mice [23] (Jax stock number 017351) were provided by Irving Weissman (Stanford University). Genotyping of the Bmi1-GFP mice was performed using genomic DNA samples. Genomic DNA isolation was performed using the DirectPCR-EAR reagent (Peqlab, Erlangen, Germany) and proteinase K (Qiagen, Hilden, Germany). Genotyping primers were purchased from Eurofins MWG Operon (Ebersberg, Germany). Separate PCR protocols were performed for the wildtype and mutant alleles. The following primer sequences were used: 1) Common: GAGAATCCAGCTGTCCAGTGT; 2) Mutant Rev: GACACGCTGAACTTGTGGCCGTTTA; and 3) Wildtype Rev: TACCCTCCACACAGGACACA.

PCRs were performed using PuReTaq Ready-To-Go^™^ PCR Beads (GE Healthcare Europe GmbH, Freiburg, Germany) according to the manufacturer’s protocol.

### Tissue harvest and fixation

To determine the Bmi1 expression pattern in the mouse cochlea, mice were sacrificed at the following time points: 1) embryonic day (E)13.5: the approximate time of the terminal mitosis of OC progenitors in mice [5]; 2) postnatal day (p)0: when the OC undergoes differentiation but is immature; and 3) p28: when the OC is functionally mature (n≥4 animals for all time points). Timed breedings were initiated, and the females were inspected the next morning for the presence of a vaginal plug; this day was then regarded as embryonic day 0.5 (E0.5). Individual embryo whole heads were fixed in 2% paraformaldehyde solution (PFA, Carl Roth GmbH, Karlsruhe, Germany) for 2 hours at 4°C. For early postnatal mice, half-heads were fixed in 2% PFA for 2 hours at 4°C. Mice aged p14 and older were anesthetized with CO_2_ before being euthanized. For the fixation of the cochlea, the entire inner ear was excised from the temporal bone and fixed by perfusing the perilymphatic fluid spaces with 4% PFA through open round and oval windows using a 30-gauge needle syringe (Microlance^®^, BD Biosciences, San Jose, CA, USA). The cochleae were then incubated in 4% PFA for 2 hours at 4°C.

### Decalcification, cryoembedding and cryosectioning

For mice aged p7 and older, the fixed cochleae were decalcified in 2 mM ethylenediaminetetraacetic acid (EDTA, Sigma-Aldrich, St. Louis, MO, USA) for 24 hours at 4°C. For cryoprotection, the heads or the isolated cochleae were incubated in 5%, 10%, 15%, and then 20% sucrose (Merck-Millipore) in 1x PBS for 1 hour per dilution. The specimens were kept at 4°C overnight in 30% sucrose in 1x PBS. The following day, the heads or the isolated cochleae were transferred into a cylindrical vessel made of aluminum foil, which was then filled with Tissue-Tek^®^ OCT^™^ Compound (Sakura Finetek, Zoeterwoude, The Netherlands) and stored at -80°C. Cryosections were prepared at a 12-μm thickness using a cryostat (Leica CM3050, Leica Biosystems, Wetzlar, Germany). Sections were placed on SuperFrost^®^ Plus microscope slides (Langenbrinck, Emmendingen, Germany), left to dry for 60 minutes at room temperature and then stored at -80°C.

### Immunolabeling and microscopy

The Bmi1 expression pattern was investigated by Bmi1 immunohistochemistry and using the Bmi1-GFP reporter mouse line, in which exon 2 of the native Bmi1 sequence has been replaced with GFP, resulting in a null allele [23]. For Bmi1 immunohistochemistry, Bmi1^WT/WT^ (Wildtype, WT) sections were labeled with a monoclonal anti-Bmi1 antibody, with retinal sections used as a positive control (data not shown), as previously described [26]. For the Bmi1-GFP reporter mouse line, Bmi1^GFP/WT^ (Heterozygous, Het) sections were labeled with an anti-GFP antibody, with duodenum sections used as a positive control (data not shown), as previously described [27]. Bmi1^GFP/GFP^ animals do not show Bmi1 expression [23] and were regarded as Bmi1 knockouts (KO) and used as a negative control for immunohistochemistry. Antigen retrieval was performed by heating slides in sodium citrate buffer (pH 6.0) for 5 minutes using a steam cooker (only for Bmi1 immunohistochemistry). The slides were allowed to cool for 5 minutes, were then permeabilized with 0.1% Triton X 100 (Sigma-Aldrich, St. Louis, MO, USA) in 1x PBS and were subsequently incubated with 5% normal donkey serum (NDS, Sigma-Aldrich) in 1× PBS for 30 minutes to block non-specific binding. When a mouse primary antibody was used, an additional blocking step was performed with the Mouse on Mouse (M.O.M.^™^) Blocking Reagent (MKB-2213, Vector Laboratories, Burlingame, CA, USA) for 1 hour at room temperature to block fluorescence caused by native mouse IgG. Primary antibodies were incubated overnight at 4°C, and secondary antibodies were incubated for 1 hour at room temperature. Counter-staining was performed using the nuclear dye 4′,6-diamidino-2-phenylindol (DAPI, Molecular Probes–Thermo Fisher Scientific, Waltham, MA, USA). Finally, slides were mounted with glass cover slips (R. Langenbrinck) using FluorSave^™^ mounting medium (Calbiochem–Merck, Darmstadt, Germany) and were stored in the dark at 4°C until microscopic analysis. A list of primary and secondary antibodies is provided in S1 Table. Fluorescence microscopy was performed using an Axio Imager M2 with an ApoTome.2 unit (Zeiss AG, Göttingen, Germany).

### RNA isolation, cDNA synthesis and quantitative real-time PCR (qRT-PCR)

Bmi1 and p16^ink4a^ mRNA levels in the OC were quantified in cochlear sensory epithelium specimens of C57Bl/6 mice at seven developmental time points: E13.5, p0, p4, p7, p14, p21 and p28. At each developmental stage, 2 independent samples were analyzed (each sample contained 4 pooled OC specimens). For the analysis of individual OC specimens of different genotypes, WT, Het and KO OC samples were harvested at p0, and analyzed independently (each sample contained two pooled OC specimens from a single mouse). For the OC-derived spheres, WT, Het and KO OC samples were cultured using the otosphere assay for 5 days *in vitro* (DIV) (see below), after which the generated spheres were harvested and analyzed independently (each sample contained 2000–3000 spheres obtained from two ears of a single mouse). After tissue micro-dissection, the samples were immediately placed into the lysis buffer of the RNAqueous^®^-Micro Kit (AM1931) (Ambion, Austin, TX, USA). RNA isolation was performed using the same kit. Complementary DNA (cDNA) synthesis was performed using a Transcriptor High Fidelity cDNA Synthesis Kit (05081955001, Roche Diagnostics, Mannheim, Germany) according to the manufacturer’s protocol. Transcript levels were measured with the Quant-iT^™^ assay on a Qubit^™^ Quantitation Platform (Thermo Fisher Scientific). mRNA levels were measured using qRT-PCR. For each qRT-PCR reaction, the cDNA level was adjusted to 5 ng in a total volume of 20 μl, and the reaction was performed using a LightCycler^®^ 480 Probes Master Mix (04707494001, Roche Diagnostics) according to the manufacturer’s protocol. Hprt, Tbp, Ubc and Gapdh were used as housekeeping genes. Bmi1, Hprt, Tbp, Ubc, Gapdh, Caspase-3 and Caspase-9 probes were designed by RealTime Ready Single Assays (Roche Applied Science) with the following Assay IDs: Bmi1 (311828), Hprt (307879), Tbp (300314), Ubc (311816), Gapdh (307884), Caspase-3 (300362) and Caspase-9 (300366). For detecting p16^ink4a^ mRNA, a FAM-conjugated TaqMan probe was purchased from TIB Molbiol GmbH (Berlin, Germany) and was used in combination with the following primers: p16-Forward, GGTCGTACCCCGATTCAGGT and p16-Reverse, TCGAATCTGCACCGTAGTTGAG. The C_T_ values were determined using LightCycler^®^ 480 Software version 1.5.0 SP4 (Roche Diagnostics), and the relative quantification was calculated using the formula 2^-ΔΔC^_T_ [28]. Measurements were conducted in triplicate for each sample.

### Otosphere assay

The OCs of p0 Bmi1^WT/WT^ (WT), Bmi1^GFP/WT^ (Het) and Bmi1^GFP/GFP^ (KO) littermates were micro-dissected and cultured, as previously described [4, 20]. Two OCs from each animal were grouped together and considered as one specimen. Briefly, the OC specimens were incubated with 0.25% trypsin / EDTA (Sigma-Aldrich) for 15 minutes at 37°C. Enzymatic digestion by trypsin was stopped by the addition of a trypsin inhibitor (Serva, Heidelberg, Germany) and DNase I (Worthington, Lakewood, NJ, USA), and the cells were mechanically dissociated by trituration. Cells were counted using a Neubauer chamber and were seeded into 10 ml Petri dishes (Greiner Bio-one GmbH, Frickenhausen, Germany) containing medium supplemented with growth factors, as previously described [20]. The culture medium was composed of Dulbecco's Modified Eagle Medium (DMEM)/ F12 media (mixed 1:1), with N2 and B-27 supplements (Gibco^®^, Thermo Fisher Scientific) and ampicillin (100 μg/mL, Sigma-Aldrich), in addition to the growth factors bFGF (10 ng/mL, R&D Systems, Minneapolis, MN, USA) and IGF-1 (50 ng/mL, R&D Systems). After 5 days *in vitro* (5DIV), the spheres were visually counted under an inverted microscope with a 20x objective (Zeiss AG). After sphere counting, 10 μM 5-ethynyl-2'-deoxyuridine (EdU) (Thermo Fisher Scientific) was added to the culture medium for an additional 24 hours. EdU is a synthetic thymidine analogue that is incorporated during DNA synthesis in proliferating cells and, thus, is used as a marker for the S-phase of the cell cycle. At 6DIV, the sphere suspension was transferred to 8-well slides (BD Biosciences) that were pre-coated with 10% Matrigel^®^ (Growth Factor Reduced, BD Biosciences). The spheres were then fixed with 2% PFA for 15 minutes at 4°C. Immunolabeling was performed for Ki67, which marks all active phases of the cell cycle [29], and phospho-Histone H3 (pHH3), a marker of the M-phase [30]. EdU labeling was performed according to the manufacturer’s instructions. The number of cells per sphere was determined by counting the DAPI-labeled nuclei. EdU-, Ki67- or pHH3-positive cells were counted within the spheres of each genotype. For every marker, 100 spheres (50 spheres x 2 animals) were analyzed per group.

### Viral transduction of the otosphere cultures

The OC specimens of C57Bl/6 mice were harvested at p0–p2. The OC specimens were dissociated, and the cell suspension was cultured in medium supplemented with growth factors, as described above. Two viral vectors (Vector BioLabs, Malvern, PA, USA) were used to transduce the cells in the otosphere cultures. Both vectors are recombinant adenoviruses with deletions in the E1 and E3 regions (Type 5 dE1/E3), rendering them replication-defective. To overexpress p16^ink4a^, an adenovirus was used to express both p16^ink4a^ and GFP, each under a separate cytomegalovirus (CMV) promoter (Ad-p16-GFP). The co-expression of p16^ink4a^ and GFP enabled to mark the transduced cells, which overexpress p16^ink4a^, by visualizing GFP fluorescence. As a control, the cells were incubated with another adenoviral vector, which expresses GFP under the CMV promoter (Ad-GFP).

For the viral transduction experiments, the cells were incubated for 6DIV in 6-well multiwell plates with a cell-repellant surface (CELLSTAR^®^, Greiner Bio-one GmbH), with either the Ad-GFP or Ad-p16-GFP vectors. Each well contained 1 ml of medium, seeded with 50000 OC-derived cells. Both vectors were added at a Multiplicity of Infection (MOI) value of 100. After 5DIV, the spheres were counted by visual inspection under an inverted microscope with a 20x objective (Zeiss AG). At 5DIV, EdU (final concentration 10 μM) was added to the culture media for the last 24 hours of culture. At 6DIV, the spheres were fixed and labeled for DAPI and EdU. GFP fluorescence was directly observed under a fluorescence microscope (ApoTome.2, Zeiss AG), and the percentage of GFP-positive cells was considered as the transduction efficiency of both vectors. Under our culture conditions, the average transduction efficiency in the spheres was 28% for the Ad-GFP vector, and 32% for the Ad-p16-GFP vector.

### Statistical analysis

qRT-PCR data and results from the otosphere assay were assessed using JMP (Version 9; SAS Institute, Cary, NC, USA). Statistical comparisons were conducted relative to the biological reference group. For all experiments, the differences between experimental groups were considered statistically significant at p < 0.05. Normally distributed data were analyzed using either the Student’s t-test for the comparison of two groups, or one-way ANOVA followed by Tukey’s post-hoc test for multiple comparisons. For the quantification of cell cycle markers in otospheres, zero values were frequently obtained, which rendered the data distribution non-normal. For these experiments, non-parametric statistical testing was performed (the Dwass-Steel test for multiple comparisons).

## Results

### Bmi1 is expressed in the mouse inner ear

**Fig 1:**
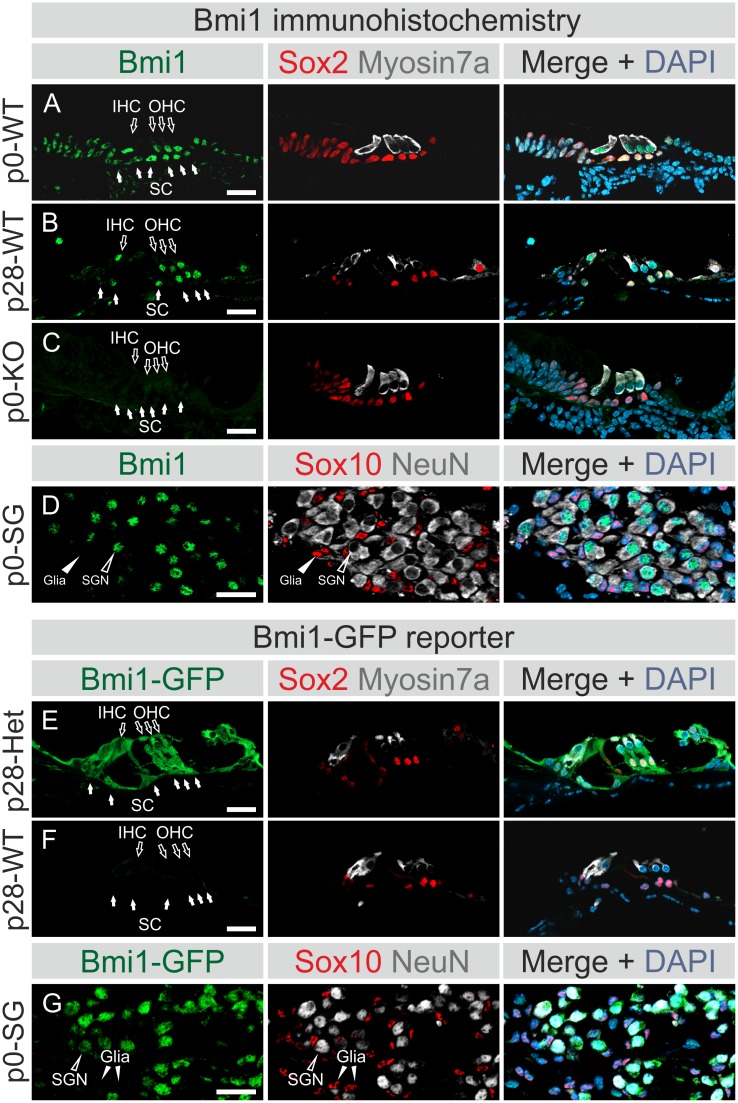
Bmi1 expression in the cochlear sensory epithelium. **(A, B, and D)** Immunohistochemical staining was performed using an anti-Bmi1 antibody on wildtype mice (Bmi1^WT/WT^, referred to as WT). **(E and G)** Alternatively, immunolabeling was performed using an anti-GFP antibody on Bmi1-GFP heterozygous mice (Bmi1^GFP/WT^, referred to as Het). OC sections **(A-C, E and F)** were co-stained for Sox2 (red) and Myosin7a (white) as markers of supporting cells and hair cells, respectively. Hair cells are marked by hollow white arrows, and supporting cells are indicated by solid white arrows. Spiral ganglion sections **(D and G)** were co-labeled with Sox10 (red) and NeuN (white) to serve as markers of glial and neuronal cells, respectively. Spiral ganglion neurons are labeled with hollow white arrowheads, and glial cells are marked with solid white arrowheads. **(A)** Bmi1 is expressed in the immature OC at p0. Both hair cells and supporting cells were labeled for Bmi1. **(B)** Bmi1 expression was detected in hair and supporting cells of the functionally mature OC at p28. **(C)** Cochleae of homozygous Bmi1-GFP mice (Bmi1^GFP/GFP^, referred to as KO) served as a negative control for Bmi1 immunohistochemistry. **(D and G)** Bmi1 expression co-localized with the neuronal marker NeuN in spiral ganglion neurons. Sox10-positive glial cells did not show Bmi1 expression. **(E)** Bmi1-GFP signal was observed in hair and supporting cells of the OC at p28. **(F)** Cochleae of WT mice served as a negative control for the Bmi1-GFP signal. Nuclei were labeled with DAPI in all sections. Solid white arrows indicate supporting cells, while hollow arrows point to inner and outer hair cells. IHC: inner hair cells, OHC: outer hair cells, SC: supporting cells, SG: spiral ganglion, SGN: spiral ganglion neurons. WT: wildtype, Het: heterozygous, KO: knockout. Scale: 20 μm.

Bmi1 expression was analyzed in the cochlea of Bmi1^WT/WT^ (WT) mice via Bmi1 immunohistochemistry (Fig 1A and 1B) and in the OC of Bmi1^GFP/WT^ (Het) mice via GFP immunolabeling (Fig 1E). Bmi1 expression was investigated at E13.5, p0 and p28. To characterize Bmi1-expressing cells, Myosin7a was used as a marker for hair cells [31] (Fig 1A–1C, 1E and 1F; shown in white), and Sox2 was used as a marker for supporting cells [32] (Fig 1A–1C, 1E and 1F; shown in red). At E13.5, Bmi1 expression was detected in the cochlear duct epithelium and spiral ganglion, with weaker expression in the surrounding otic mesenchyme (data not shown). Specific nuclear Bmi1 staining was detected in hair cells and supporting cells at p0 (Fig 1A) and p28 (Fig 1B). As a negative control, no specific Bmi1 signal was detected in the Bmi1^GFP/GFP^ (Knockout; KO) OC (shown at p0, Fig 1C). Bmi1 expression was also detected in the spiral ganglion (shown at p0, Fig 1D). Co-labeling with the neural marker NeuN and the glial marker Sox10 revealed that the Bmi1-positive cells were spiral ganglion neurons (Fig 1D). Bmi1-GFP expression revealed a pattern similar to that of the native Bmi1 protein at all time points (Fig 1E and 1G; data not shown). At p28, Bmi1-GFP expression was observed in hair cells and supporting cells of the OC (Fig 1E). Bmi1-GFP expression was not seen in the negative control WT cochleae (Fig 1F).

**Fig 2:**
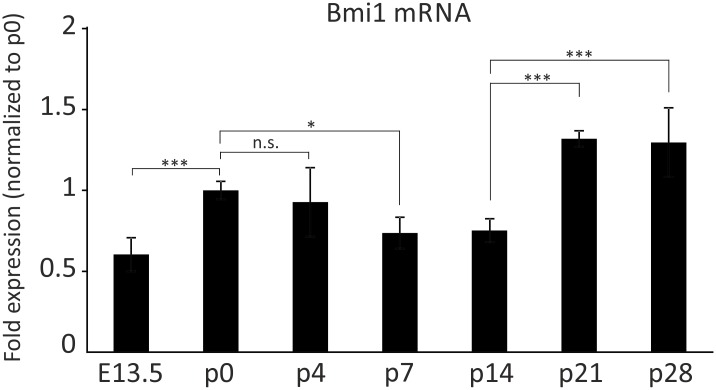
Quantitative analysis of the temporal Bmi1 expression pattern in the cochlear sensory epithelium during development. Bmi1 mRNA levels in the cochlear sensory epithelium at seven developmental stages: E13.5, p0, p4, p7, p14, p21 and p28. Bmi1 transcripts were detected in the sensory epithelium at all stages. All values were normalized to the p0 level. Bmi1 mRNA levels significantly increased between E13.5 and p0. Subsequently, Bmi1 mRNA was significantly downregulated between p0 and p7, which was followed by a statistically significant upregulation at p21 and p28. n.s.: not significant. *p<0.05, ***p<0.001.

Bmi1 protein expression was also observed in hair cells and supporting cells of the neonatal utricle, ampulla and saccule (S1 Fig) at p0. Using qRT-PCR, Bmi1 mRNA was detected in the cochlear epithelium at E13.5, p0, p4, p7, p14, p21 and p28 (n = 2 independent samples each) (Fig 2). Bmi1 mRNA levels were significantly upregulated between E13.5 and p0 (one-way ANOVA followed by Tukey’s post-hoc test, p<0.001) and were then significantly downregulated between p0 and p7 (one-way ANOVA followed by Tukey’s post-hoc test, p<0.05) (Fig 2). Bmi1 mRNA levels were similar at p7 and p14. This was followed by a statistically significant upregulation in the functionally mature OC at p21 and p28 (p<0.001) (Fig 2).

In summary, we confirmed previous reports of Bmi1 expression in the embryonic [20] and postnatal [21] cochlear sensory epithelia. Additionally, we described the temporal mRNA expression pattern of Bmi1 and revealed the novel expression of Bmi1 protein in spiral ganglion neurons and vestibular sensory epithelia.

### Bmi1 contributes to otic progenitor cell proliferation *in vitro*

**Fig 3:**
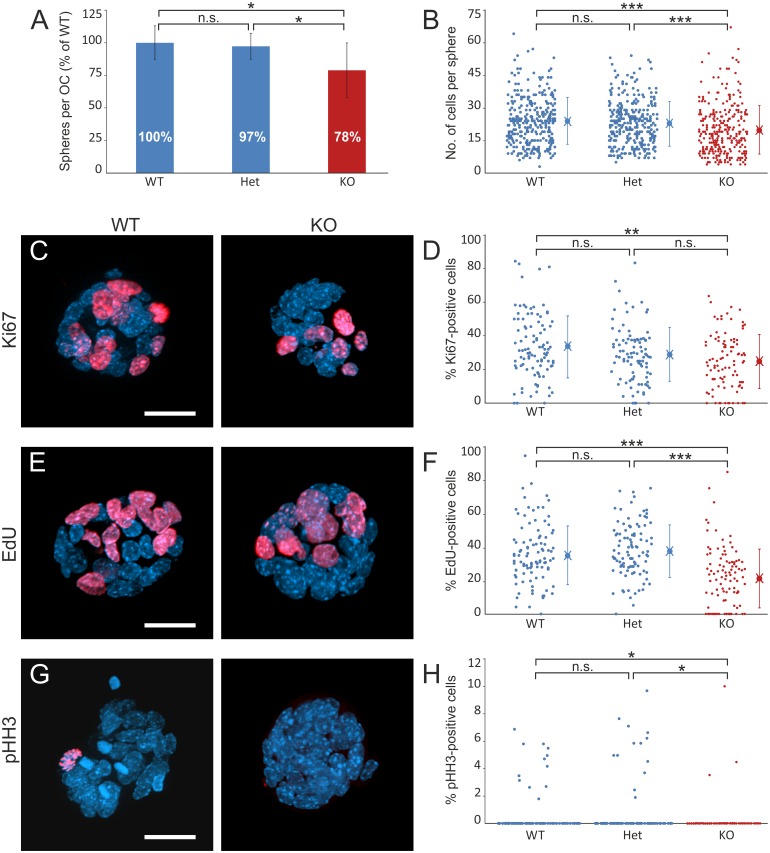
Sphere-forming capacity of the neonatal Bmi1 knockout organ of Corti. **(A)** Average number of spheres per OC, expressed as a percentage of the WT control (mean ± standard deviation). The data represent the results from a total of 9 WT, 19 Het and 6 KO p0 mice pooled from 4 independent experiments. Cultures from the KO mice generated significantly fewer spheres compared to their WT littermates (one-way ANOVA followed by Tukey‘s post-hoc test, p<0.05). The difference between the WT and Het mice is not significant. (**B, D, F and H**) Quantification of DAPI, Ki67, EdU and pHH3. Each data point represents one sphere. Mean and standard deviation values are shown in the dot plots. **(B)** Number of DAPI-labeled cells per sphere. The number of cells per sphere is significantly reduced for the Bmi1 KO spheres compared to the WT spheres (p<0.001, Dwass-Steel test; n = 300 spheres per group). The difference between WT and Het mice is not statistically significant. (**C, E and G**) Representative images of the WT and KO spheres, labeled for Ki67 (**C**), EdU (**E**) and pHH3 (**G**). (**D**) Percentage of Ki67-positive cells in the spheres: the KO spheres harbor a significantly lower percentage of Ki67-positive cells compared with the WT spheres (p<0.01, Dwass-Steel; n = 100 spheres per group). There was no significant difference between the WT and Het mice, or between Het and KO mice. (**F**) Percentage of EdU-incorporating cells in the spheres: the KO spheres contain a significantly lower percentage of EdU-positive cells compared with the WT spheres and Het spheres (p<0.001, Dwass-Steel test; n = 100 spheres per group). The difference between the WT and Het mice is not significant. (**H**) Percentage of pHH3-positive cells in the spheres: the percentage of pHH3-expressing cells is significantly lower in the KO spheres compared with the WT spheres (p<0.05, Dwass-Steel test; n = 100 spheres per group). There was no statistically significant difference between the WT and Het spheres. WT: wildtype, Het: heterozygous, KO: knockout. *p<0.05, **p<0.01, ***p<0.001.

To determine whether Bmi1 influences otic progenitor cell proliferation *in vitro*, the OC of WT, Het and KO p0 animals were cultured using the otosphere assay, as described in the Methods section. After 5 days *in vitro* (5DIV), the spheres were counted, and proliferating cells were detected by Ki67, EdU or phospho-Histone H3 (pHH3) labeling and manual counting. The average numbers of cells per OC were 130,833 (± 20,766) for WT animals (n = 9), 127,828 (± 14,941) for Het animals (n = 19) and 112,500 (± 20,493) for KO animals (n = 6). There was no statistically significant difference in the number of cells per OC across groups (one-way ANOVA). At 5DIV, the average numbers of spheres per OC were 1599.61 (± 415.47) for WT animals, 1368.92 (± 149.02) for heterozygous animals and 1233.41 (± 215.53) for KO animals. To account for inter-experimental variability in sphere numbers and genotype frequencies, all values from one experiment were normalized to the average WT control of the corresponding experiment. When normalized to the WT control, the Het mice (n = 19) gave rise to 97.3% (± 10%), and the KO mice (n = 6) gave rise to 78.89% (± 21%), the latter of which is significantly lower than the WT control (n = 9) (one-way ANOVA followed by Tukey’s post-hoc test, p<0.05) (Fig 3A). To determine whether this decrease in sphere-forming capacity is related to reduced cell proliferation, the spheres from all genotypes were fixed and labeled using Ki67, EdU and pHH3 markers. As determined by the DAPI signal, the mean number of cells per sphere was 19.78 (± 11.3) for the KO spheres, which was significantly lower than the mean numbers for the WT (23.96 ± 11.01) and Het spheres (22.75 ± 10.2) (p<0.001, Dwass-Steel test; n = 300 spheres analyzed per group) (Fig 3B). The KO spheres contained a mean of 24.71% (± 16.23%) Ki67-positive cells, which was significantly lower than the mean of WT spheres (33.63% ± 18.77%) (p<0.01, Dwass-Steel test) but not significantly different from the Het spheres (28.94% ± 16.28%) (p>0.05, Dwass-Steel test; n = 100 spheres analyzed per group) (Fig 3C and 3D). Furthermore, the percentage of EdU-incorporating cells was significantly reduced in the KO spheres (22.17% ± 17.65%) compared with the WT spheres (35.99% ± 17.22%) and the Het spheres (38.51% ± 15.82%) (p<0.001, Dwass-Steel test; n = 100 spheres analyzed per group) (Fig 3E and 3F). We observed a statistically significant reduction in the percentage of pHH3-positive cells in the KO spheres (0.18% ± 1.50%) compared with the WT (0.51% ± 1.1%) and Het spheres (0.71% ± 2%) (p<0.05, Dwass-Steel; n = 100 spheres analyzed per group) (Fig 3G and 3H). There was no statistically significant difference between the WT and Het spheres for any of the abovementioned parameters.

These findings suggest that Bmi1 contributes to the sphere-forming capacity of the OC and to otic progenitor cell proliferation.

### Bmi1 is not essential for the morphological development of the OC *in vivo*

**Fig 4:**
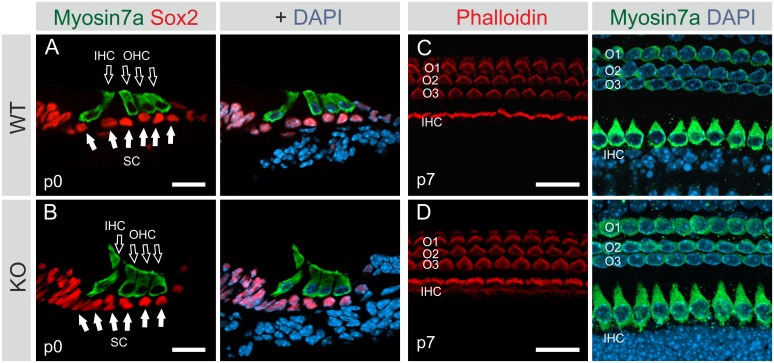
Phenotype of the early postnatal Bmi1 knockout organ of Corti. **(A and B)** Sections of the cochleae of WT **(A)** and KO **(B)** mice at p0, immunolabeled with Myosin7a (green) and Sox2 (red) and counterstained with DAPI (blue). Hollow white arrows mark inner and outer hair cells. Solid white arrows label supporting cells. Immunohistochemical analysis shows normal development of the Bmi1 KO OC, as determined by the expression of the hair cell marker Myosin7a and the supporting cell marker Sox2. **(C and D)** Surface views of the OC of WT **(C)** and KO **(D)** mice at p7, stained with phalloidin, which labels stereocilia, and Myosin7a, a hair cell marker. Nuclei were labeled with DAPI. Immunohistochemical analysis shows normal development of the Bmi1 KO OC. Both WT and KO mice possess three rows of outer hair cells (O1, O2 and O3) and one row of inner hair cells. Neither WT nor KO OC show disorganized structure or stereocilia abnormalities at p7. WT: wildtype, KO: knockout, IHC: inner hair cells, OHC: outer hair cells, SC: supporting cells. Scale: 20 μm.

To determine whether Bmi1 loss affects the morphological development of the OC, we performed immunolabeling of cochlear sections and whole mount preparations at p0 and p7. There were no gross abnormalities detected in the architecture of the OC at either p0 or p7 (three KO animals analyzed at each stage) (Fig 4A–4D). At p0, Myosin7a was observed in hair cells, and Sox2 was observed in supporting cells, both in patterns similar to those in the WT OC (Fig 4A and 4B). The analysis of KO mid-modiolar cochlear sections at p0 revealed that all of the cochlear turns formed normally and were identical to those of the WT cochlea (S2 Fig). At p7, the KO OC displayed three rows of outer hair cells and one row of inner hair cells, similar to the WT OC (Fig 4C and 4D). In the Bmi1 KO OC, the hair cells were characterized by intact stereocilia, which did not show signs of disorganization (Fig 4C and 4D).

These findings suggest that Bmi1 is not essential for the embryonic and early postnatal development of the OC.

### Bmi1 loss is associated with p16^ink4a^ upregulation in otic progenitor cells *in vitro*

To better understand the molecular changes that occur upon Bmi1 loss in the OC and otospheres, we investigated Bmi1 and p16^ink4a^ gene expression changes in the neonatal OC and otospheres of different genotypes, by immunohistochemistry and qRT-PCR.

**Fig 5:**
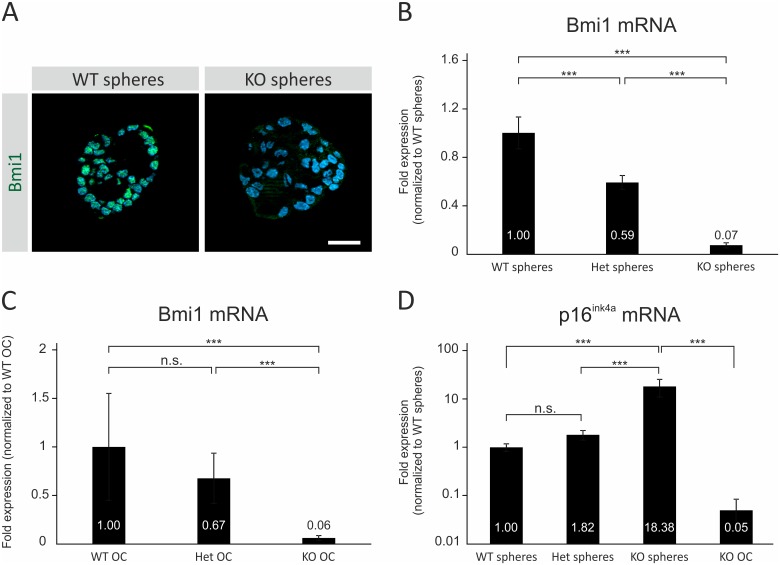
Gene expression changes in the Bmi1 knockout organ of Corti and otospheres. **(A)** Representative high-magnification images of Bmi1 WT and KO spheres after 5DIV, stained for Bmi1 (green) and counterstained with DAPI (blue). Nuclear Bmi1 signal was detected in the WT, but not the KO, spheres. **(B)** Quantitative analysis of Bmi1 mRNA levels in the WT, Het and KO spheres by qRT-PCR. The Het spheres showed Bmi1 mRNA levels that are 0.59-fold relative to the WT spheres. Limited amounts of mRNA were detected in the KO spheres, representing 0.07-fold compared with the WT control. **(C)** Quantitative analysis of Bmi1 transcript levels in the neonatal WT, Het and KO OC by qRT-PCR. When normalized to the WT control, the Bmi1 mRNA levels in the Het OC were 0.67-fold of the WT level. In the KO OC, the level of mRNA detected was 0.06-fold of the WT expression level. **(D)** Quantitative analysis of p16^ink4a^ transcript levels in the WT, Het and KO spheres by qRT-PCR. In the Het spheres, p16^ink4a^ is upregulated 1.82-fold relative to the WT spheres. The KO spheres show an 18.38-fold upregulation of p16^ink4a^ compared to the WT spheres. In the neonatal KO OC, only scarce amounts of p16^ink4a^ mRNA were detected, representing 0.05-fold compared to the WT spheres. WT: wildtype, Het: heterozygous, KO: knockout. n.s.: not significant. ***p<0.001.

Bmi1 protein expression was detected immunohistochemically in the WT spheres after 5DIV but not in the KO spheres (Fig 5A). qRT-PCR analysis revealed that Bmi1 mRNA levels in the Het spheres (n = 3 samples) were 0.59-fold of the WT level (n = 3 samples) (Fig 5B). In the KO spheres (n = 2 samples), the level of mRNA detected was 0.07-fold of the WT expression level (Fig 5B). When the neonatal OC was harvested at p0, Bmi1 mRNA levels were 0.67 fold in the Het OC (n = 3), and 0.06 fold in the KO OC (n = 4), compared to the WT control (n = 3) (Fig 5C).

We next asked whether p16^ink4a^ mRNA was expressed in the OC during cochlear development. P16^ink4a^ mRNA was not detected in the WT OC at any time point from E13.5 until p28, our latest time point (n = 2 independent samples per time point, data not shown). An adult mouse spleen served as a positive control for p16^ink4a^ expression. To determine whether p16^ink4a^ expression is related to the Bmi1 expression level in otospheres, qRT-PCR was used to measure p16^ink4a^ mRNA levels in the WT (n = 3 samples), Het (n = 3 samples), and KO spheres (n = 2 samples). When normalized to the WT control, the Het spheres showed a 1.8-fold upregulation of p16^ink4a^, which was not a statistically significant difference but showed a trend towards significance (Fig 5D) (one-way ANOVA followed by Tukey’s post-hoc test, p = 0.087). The KO spheres showed an 18.3-fold upregulation of p16^ink4a^ mRNA, which was statistically significant compared with both the WT and Het spheres (Fig 5D) (one-way ANOVA followed by Tukey’s post-hoc test, p<0.001). With regard to p16^ink4a^ mRNA expression in the neonatal OC, p16^ink4a^ mRNA was not detected in the WT (n = 3) or Het OC (n = 3) at p0, and was only detected in the KO OC (n = 4), albeit in scarce amounts, representing 0.05-fold of the mRNA level in the WT spheres (Fig 5D).

These data indicate that p16^ink4a^ mRNA is not expressed in the developing WT OC, but is expressed in scarce amounts in the KO OC. In contrast, p16^ink4a^ upregulation was observed in WT otic progenitor cells *in vitro* and to a greater extent, in KO otic progenitors. This discrepancy may suggest that p16^ink4a^ expression in the cells of the OC, is more tightly repressed *in vivo* than *in vitro*.

### P16^ink4a^ overexpression results in a reduced sphere-forming capacity of the organ of Corti

**Fig 6:**
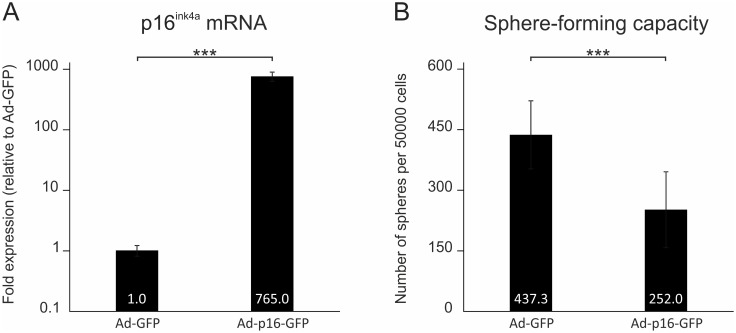
Effect of viral vector-mediated p16^ink4a^ overexpression on the sphere-forming capacity of the organ of Corti. **(A)** Quantitative analysis of p16^ink4a^ mRNA levels in otospheres, derived from wildtype organ of Corti specimens after 5 days *in vitro*. The organ of Corti-derived cells were incubated with either of two viral vectors: i) Ad-GFP to induce the expression of GFP, or Ad-p16-GFP to induce the expression of both GFP and p16^ink4a^. The spheres incubated with Ad-p16-GFP showed a 765-fold increase in p16^ink4a^ mRNA levels compared to the spheres incubated with Ad-GFP (n = 2 independent samples, measured in triplicate, for both groups). The difference in p16^ink4a^ mRNA levels between the Ad-GFP and Ad-p16-GFP groups was highly statistically significant (Student’s t-test, p<0.001). **(B)** Average number of spheres generated after 5 days *in vitro* per 50000 cells plated (mean ± standard deviation). Cells incubated with Ad-p16-GFP generated significantly fewer spheres, after 5 days *in vitro*, compared to the cells incubated with Ad-GFP (N = 3 independent experiments, n = 5 replicates per experiment, Student’s t-test, p<0.001). ***p<0.001.

To determine whether p16^ink4a^ overexpression in OC-derived cells is sufficient to inhibit their sphere-forming capacity, OC specimens of WT mice were harvested, dissociated, and cultured using the otosphere assay. The cells were incubated with either of two viral vectors: i) an adenovirus expressing GFP under the cytomegalovirus (CMV) promoter (Ad-GFP), or ii) an adenovirus expressing p16^ink4a^ and GFP, each under a separate CMV promoter (Ad-p16-GFP). After 5DIV, the spheres, which were generated *in vitro*, were counted for both groups. To confirm p16^ink4a^ overexpression by the Ad-p16-GFP vector, qRT-PCR was used to quantify p16^ink4a^ mRNA levels in the spheres incubated with either vector. The spheres, which were incubated with the Ad-p16-GFP vector showed a significant 765-fold increase in p16^ink4a^ mRNA levels, compared to the spheres incubated with Ad-GFP (Fig 6A) (n = 2 independent samples measured in triplicate, for both vectors, Student’s t-test, p<0.001). These data indicate that otic progenitor cells can be successfully transduced with Ad-p16-GFP, and that the transduction of the cells by this vector results in a significant upregulation of p16^ink4a^. We next investigated the effect of p16^ink4a^ overexpression on the number otospheres generated *in vitro*. At DIV5, the average number of spheres generated per 50000 cells was 252 ± 60.5 spheres for the Ad-p16-GFP vector, which was significantly lower than that for the Ad-GFP vector (437.3 ± 93.6 spheres) (Fig 6B) (N = 3 independent experiments, n = 5 replicates per experiment, Student’s t-test, p<0.001). To gain insight into the mechanism of p16^ink4a^-mediated inhibition of sphere formation, we added EdU to the culture media during the last 24 hours of culture, and performed EdU labeling for both groups. We could not detect a significant difference in the percentage of EdU-incoporating cells among both groups, which may be related to the finding that the control vector itself (Ad-GFP) reduced EdU incorporation and led to a significant upregulation of p16^ink4a^ in these experiments (data not shown). Accordingly, we tested whether p16^ink4a^ overexpression may induce apoptosis in the spheres. No difference was detected between both groups with regard to the transcript levels of the apoptosis-related genes caspase-3 and caspase-9, as analyzed by qRT-PCR (S3 Fig). This result may suggest that the reduction in sphere numbers, observed with p16^ink4a^ overexpression, is not related to increased apoptosis.

These findings indicate that viral vector-mediated p16^ink4a^ overexpression results in a decrease of the sphere-forming capacity of the OC. While the elucidation of the mechanism behind p16^ink4a^-mediated inhibition of sphere formation requires further detailed investigations, the findings support a link between p16^ink4a^ upregulation in the Bmi1 KO spheres, and the reduced sphere-forming capacity of the Bmi1 KO OC.

## Discussion

In the present study, Bmi1 expression was shown in the postnatal and mature OC. Bmi1 loss did not lead to an obvious phenotype or malformation of the organ by the end of the first postnatal week. In contrast, Bmi1 loss resulted in a reduced generation of otospheres and reduced expression of proliferation markers in the spheres. This phenotype was associated with p16^ink4a^ upregulation. These findings suggest that Bmi1 contributes to the maintenance of otic progenitor cell proliferation, a role which may be linked to p16^ink4a^ repression *in vitro*.

### Bmi1 is expressed in the mouse inner ear

Bmi1 expression has been extensively studied in several tissues. Notable examples include the brain [26, 33, 34], retina [26, 33, 35], and cochlea [20, 21]. In the present study, we showed Bmi1 expression in both the developing and the functionally mature OC through Bmi1 immunohistochemistry and a Bmi1-GFP reporter mouse line (Fig 1). Bmi1 expression was observed in both hair and supporting cells of the OC. We additionally observed Bmi1 expression in the neonatal utricle, crista ampullaris and saccule (S1 Fig). This finding is potentially of interest, since vestibular sensory epithelia possess a sphere-forming capacity, which persists into adulthood in mice [4, 36].

Analysis of the temporal Bmi1 expression revealed fluctuations in Bmi1 mRNA levels during cochlear development, although the fold changes were modest, so it is possible that these changes have no functional relevance (Fig 2). These results are consistent with recent RNA-Seq data reported in a gene expression analysis of the OC [37]. The finding, that Bmi1 is not downregulated between E13.5 and p28, suggests that Bmi1 plays roles in the functionally mature OC distinct from promoting proliferation, such as the described anti-apoptotic role of Bmi1 in the OC [21]. Indeed, Bmi1 expression is not unique to proliferating cells but is also detected in postmitotic cells, notably neurons [26, 33]. One function of Bmi1 in neurons is the repression of p19arf and the protection against p53-dependent apoptosis [26]. In the present study, we also demonstrated Bmi1 expression in spiral ganglion neurons (Fig 1D and 1G).

### Bmi1 contributes to otic progenitor cell proliferation *in vitro*

Our findings show that, in the absence of Bmi1, the sphere-forming capacity of the OC was reduced (Fig 3A). Bmi1 KO spheres were composed of fewer cells compared with the WT spheres (Fig 3B). This phenotype was associated with a lower percentage of cells expressing cell cycle markers *in vitro* (Fig 3C–3H). Lu and colleagues [22] described the reduced EdU incorporation in supporting cells of the cultured Bmi1 KO OC in response to neomycin-induced damage. The authors of that study also reported the reduced sphere-forming capacity of the Bmi1 KO OC, although they did not characterize the cell cycle status of the cells in the spheres [22]. In this regard, our own findings both complement and confirm the results of their study. Our findings showed the reduced expression of Ki67 in the KO spheres, which indicated a reduction in the number of cycling cells (Fig 3C and 3D). Likewise, the decreased abundance of EdU- and pHH3-positive cells indicated lower proportions of cells entering the S- and M-phases, respectively (Fig 3E–3H). Among the three markers, the largest reduction was observed for EdU incorporation, which may suggest that the primary cellular event is reduced entry into the S-phase. This explanation would be consistent with an influence of Bmi1 on the p16^ink4a^/Rb pathway, which regulates progression from the G1- to the S-phase [38]. In line with this notion, a marked upregulation of p16^ink4a^ was found in the KO spheres (Fig 5D).

### Bmi1 is not essential for the morphological development of the OC *in vivo*

Previous studies have reported that Bmi1 has a more profound effect on cell proliferation in culture than *in vivo* [8, 39]. In the present study, despite the reduced sphere-forming capacity of Bmi1 KO OC specimens, we did not detect morphological abnormalities in the early postnatal Bmi1 KO OC (Fig 4 and S2 Fig), which suggests that the proliferation capacity of cochlear progenitors was not substantially reduced during embryonic development *in vivo*. Taken together, these findings confirm the results of two recent studies of Bmi1 function in the OC [21, 22]. However, those studies did not address the question of why Bmi1 loss impacts otic progenitor cell proliferation in OC-derived spheres *in vitro* but not in the developing cochlea *in vivo*. In our study, we attempted to answer this question by comparing p16^ink4a^ expression *in vitro* versus *in vivo*. Molofsky and colleagues [8] described a reduced sphere-forming capacity of the neonatal Bmi1 KO brain, despite relatively normal brain development at birth. Similarly, Bmi1 overexpression in the central nervous system (CNS) enhanced cell proliferation and neurosphere formation *in vitro* but had only marginal effects on proliferation and stem cell frequency *in vivo* [39]. In those studies, the authors attributed those discrepancies to the fact that p16^ink4a^ and p19^arf^, Bmi1 downstream targets, are expressed in culture but not *in vivo*. P16^ink4a^ is not expressed in the brains of developing and young adult mice *in vivo* [8, 34, 39–41] but is induced in neurosphere cultures [8, 39, 41] as a stress response to the unphysiological culture environment [42, 43]. Our findings extend this phenomenon to otic progenitor cells. In our study, p16^ink4a^ mRNA was not detected in the developing OC *in vivo*. This finding is consistent with recent studies reporting the failure to detect p16^ink4a^ mRNA in the early postnatal OC by RNA-Seq [37] or single-cell qRT-PCR [44]. Interestingly, p16^ink4a^ induction occurred upon the dissociation and culture of OC cells using the otosphere assay (Fig 5D). We propose that p16^ink4a^ induction in culture renders the cells more dependent on Bmi1-mediated repression *in vitro*. Therefore, Bmi1 loss influences the proliferative capacity *in vitro* in a manner that remains unrecognized *in vivo*. Additionally, the lack of p16^ink4a^ expression in the OC *in vivo* makes it unlikely that p16^ink4a^ is involved in the depletion of otic progenitors that takes place during cochlear development.

### Bmi1 loss is associated with p16^ink4a^ upregulation in otic progenitor cells *in vitro*

Our results did not show an effect of Bmi1 haplo-insufficiency with regard to sphere-forming potential (Fig 3A). Bmi1 Het spheres showed decreased Bmi1 mRNA levels that were 0.59-fold of the wildtype level (Fig 5B). This reduced Bmi1 expression level was accompanied by a 1.8-fold p16^ink4a^ upregulation compared with the WT (Fig 5D). This p16^ink4a^ expression profile was associated with a normal sphere-forming capacity and a normal expression of proliferation markers in the Het spheres (Fig 3). In contrast, the Bmi1 KO spheres showed a considerable 18-fold upregulation of p16^ink4a^ (Fig 5D), which correlated with reduced sphere-formation and cell proliferation in the KO spheres (Fig 3). Thus, the proliferative capacity of otic progenitors appears to be linked to p16^ink4a^ mRNA levels. Bruggeman and colleagues [40] described a gene-dosage effect of ink4a/arf in Bmi1-null progenitors and concluded that a threshold exists for these two proteins, which, when exceeded, leads to an inhibition of cell proliferation. Our results are in agreement with this hypothesis and may explain the absence of a proliferation defect in our study upon the loss of one Bmi1 allele.

Although Bmi1 loss resulted in a marked p16^ink4a^ upregulation in the otospheres, it resulted in only a scarce p16^ink4a^ mRNA expression in the neonatal KO OC (Fig 5D). This discrepancy may suggest that p16^ink4a^ expression is more tightly repressed *in vivo*, possibly by other factors in addition to Bmi1. We propose that the low p16^ink4a^ mRNA expression in the neonatal Bmi1 KO OC represents a sub-threshold level that is not functionally relevant. This assumption is based on a p16^ink4a^ expression threshold and gene-dosage effect, as described by Bruggeman and coworkers [40]. Taken together, the findings further support the notion that the discrepancy between Bmi1 effects on the otospheres versus the OC, is partly because of the differential expression of p16^ink4a^ in culture versus *in vivo*. Thus, the identification of additional p16^ink4a^ regulators in future studies, may have important implications for cell cycle regulation in the OC.

### P16^ink4a^ overexpression results in a reduced sphere-forming capacity of the organ of Corti

P16^ink4a^ overexpression was previously shown to inhibit proliferation in several murine cell types [18, 45, 46]. In the present study, an adenoviral vector (Ad-p16-GFP) was used to overexpress p16^ink4a^ in OC-derived cells by more than 750-fold. Upon p16^ink4a^ overexpression, the sphere-forming capacity was reduced by 42%, compared to the control cells that were incubated with Ad-GFP. This result was similar to the effect of Bmi1 loss on the sphere-forming capacity of the OC, although the reduction was more pronounced upon forced p16^ink4a^ overexpression with Ad-p16-GFP, than that observed in the Bmi1 KO spheres. The stronger reduction of otosphere generation may be attributed to the higher upregulation of p16^ink4a^ that could be achieved using the viral vector, compared to that detected in Bmi1 KO mice. Taken together, the present findings are consistent with previous studies of neural stem cells, which reported that Bmi1 promotes neurosphere formation partly through the repression of p16^ink4a^ [8, 47]. In those studies, the co-deletion of p16^ink4a^ resulted in a partial rescue of the sphere-forming capacity of Bmi1-deficient neural stem cells [8, 47]. In conclusion, our findings support the notion that the reduced proliferation detected in the Bmi1 KO otospheres is caused, at least partially, by p16^ink4a^ accumulation in culture.

### Bmi1-mediated cell cycle regulation in otic progenitor cells

**Fig 7:**
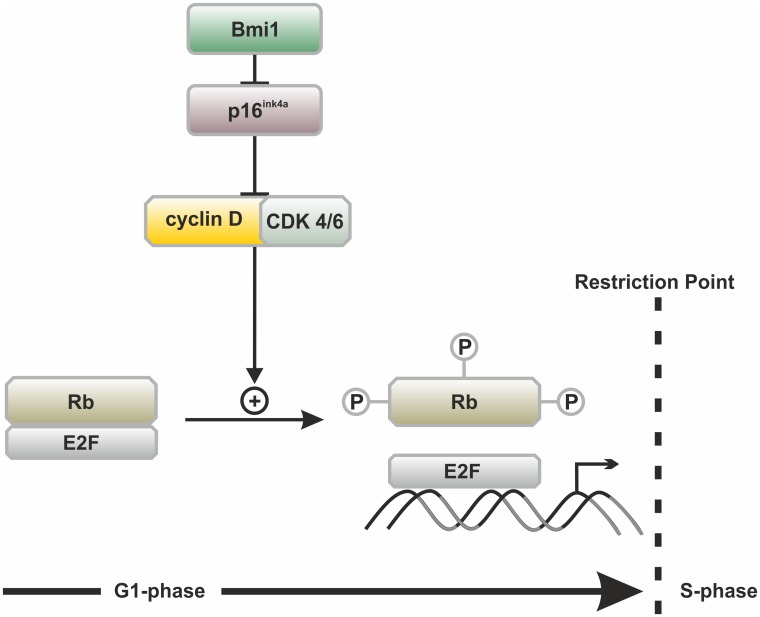
The mechanism of cell cycle regulation via the Bmi1/p16^ink4a^ pathway. Bmi1 represses p16ink4a, which in turn inhibits cyclin-dependent kinases 4/6 (CDK4/6) from binding to cyclin D. CDK4/6-cyclin D complexes are required for the phosphorylation of the retinoblastoma (Rb) family of proteins. In the absence of CDK4/6-cyclin D complexes, Rb remain in their inactive hypophosphorylated form. After phosphorylation, Rb release the elongation factor 2 (E2F) group of transcription factors. E2F activate the transcription of various genes that are required for progression from the G1-phase of the cell cycle, through the restriction point and into the S-phase. After passing the restriction point, the cell becomes committed to the cell cycle even in the absence of external proliferation stimuli. Figure adapted from [48].

Based on the finding that Bmi1 loss was accompanied by p16^ink4a^ upregulation in otic progenitor cells *in vitro* (Fig 5D), we propose a working model that may explain the reduced proliferative capacity of cultured otic progenitors upon Bmi1 loss (Fig 7). The Bmi1-mediated repression of p16^ink4a^ allows the activation of cyclin D-dependent kinases 4 and 6, which keeps the retinoblastoma (Rb) proteins in their active hyperphosphorylated form [13]. Hyperphosphorylated Rb release elongation factor 2 (E2F) transcription factors, promoting their capacity to activate genes that are required for progression through the restriction point into the S-phase of the cell cycle [14].

In the present study, Bmi1 KO spheres showed only a minor reduction in cell proliferation despite a marked p16^ink4a^ upregulation. This finding indicates that the Bmi1/p16^ink4a^ pathway is not the only cell cycle regulatory pathway active in otic progenitor cells. For example, the cyclin-dependent kinase inhibitor (CKI) p27^kip1^, which is expressed in supporting cells of the OC, appears to be downregulated upon dissociation and culture of the OC [20, 49], allowing cell proliferation to occur *in vitro* [49]. *In vivo*, it was previously shown that the CKIs, p19^ink4d^ and p27^kip1^, contribute to the active maintenance of quiescence in the cochlear hair- and supporting cells, respectively [50–53]. Deletion of p19^ink4d^ resulted in the cell cycle re-entry of the cochlear hair cells, but not supporting cells [52, 53]. In contrast, p27^kip1^-null mice showed signs of cell proliferation in supporting cells of the postnatal and adult OC *in vivo* [50, 51]. However, the number of proliferating cells in the p27^kip1^-null OC declined with age [50]. This age-dependent decline may suggest that other CKIs compensate for the absence of p27^kip1^, and inhibit cell proliferation in the adult OC. One of those candidate CKIs may potentially be p16^ink4a^, which is not expressed in the OC during development (present study). Since we did not investigate p16^ink4a^ mRNA expression in stages older than p28, it remains possible that p16^ink4a^ mRNA expression in the OC starts in adulthood. This assumption is supported by several reports, describing the increasing p16^ink4a^ mRNA expression, in different tissues, with age [16, 54, 55].

In conclusion, our findings support a role for Bmi1 as a promoter of cell proliferation in an otic progenitor cell culture, possibly through the repression of the cell cycle inhibitor p16^ink4a^. Conversely, Bmi1 was not required for the morphological development of the OC *in vivo*. Although p16^ink4a^ mRNA was not expressed in the intact WT OC, p16^ink4a^ mRNA was detected in culture. This p16^ink4a^ induction rendered OC cells more reliant on Bmi1-mediated repression in culture, a phenomenon that was previously described in brain-derived neurospheres. Taken together, our data add to the current understanding of cell cycle regulation in the OC and OC-derived progenitors, which may have implications for hair cell regeneration.

## Supporting Information

S1 Fig Bmi1 expression in neonatal vestibular sensory epithelia. **(A-C)** Sections of the vestibular apparatus of WT mice at p0, stained for Bmi1 (green) and co-labeled for Sox2 (red) and Myosin7a (white). Nuclei were labeled with DAPI. Bmi1 expression was detected in hair and supporting cells of the utricle **(A)**, crista ampullaris **(B)** and saccule **(C)**. In all three epithelia, Sox2 expression was observed in supporting cells and a subset of hair cells. Supporting cells are indicated by solid white arrows, while hair cells are indicated by hollow white arrows. SC: supporting cells, HC: hair cells. Scale: 50 μm.
(TIF)

S2 Fig Gross morphology of the Bmi1 KO OC at p0. **(A and B)** Mid-modiolar sections of the cochleae of Bmi1 WT **(A)** and KO **(B)** mice at p0, counterstained with DAPI (blue). The KO cochlea displays the normal 4–5 cochlear half-turns. All of the turns appear normally formed, with similar morphology to the WT cochlea. Scale: 200 μm.
(TIF)

S3 Fig Effect of viral vector-mediated p16^ink4a^ overexpression on the transcription of the apoptosis-related genes caspase-3 and caspase-9. **(A and B)** Quantitative analysis of caspase-3 and caspase-9 mRNA levels in organ of Corti-derived spheres, which were incubated with either of two viral vectors: i) Ad-GFP to induce the expression of GFP, or ii) Ad-p16-GFP to induce the expression of both GFP and p16^ink4a^. No significant differences were detected in the levels of caspase-3 **(A)** or caspase-9 mRNA **(B)** between the spheres incubated with Ad-GFP and those incubated with Ad-p16-GFP for 5 days *in vitro* (n = 2 independent samples, measured in triplicate, for both groups, Student’s t-test, p>0.05). n.s.: not significant.
(TIF)

S1 Table List of antibodies and fluorophores used in this study. (DOCX)
