# Supplementary figures and images for: Bmi1 Loss in the Organ of Corti Results in p16ink4a Upregulation and Reduced Cell Proliferation of Otic Progenitors In Vitro
Source: PLoS One. 2016 Oct 18;11(10):e0164579. doi: 10.1371/journal.pone.0164579 (PMC5068820; doi:10.1371/journal.pone.0164579)

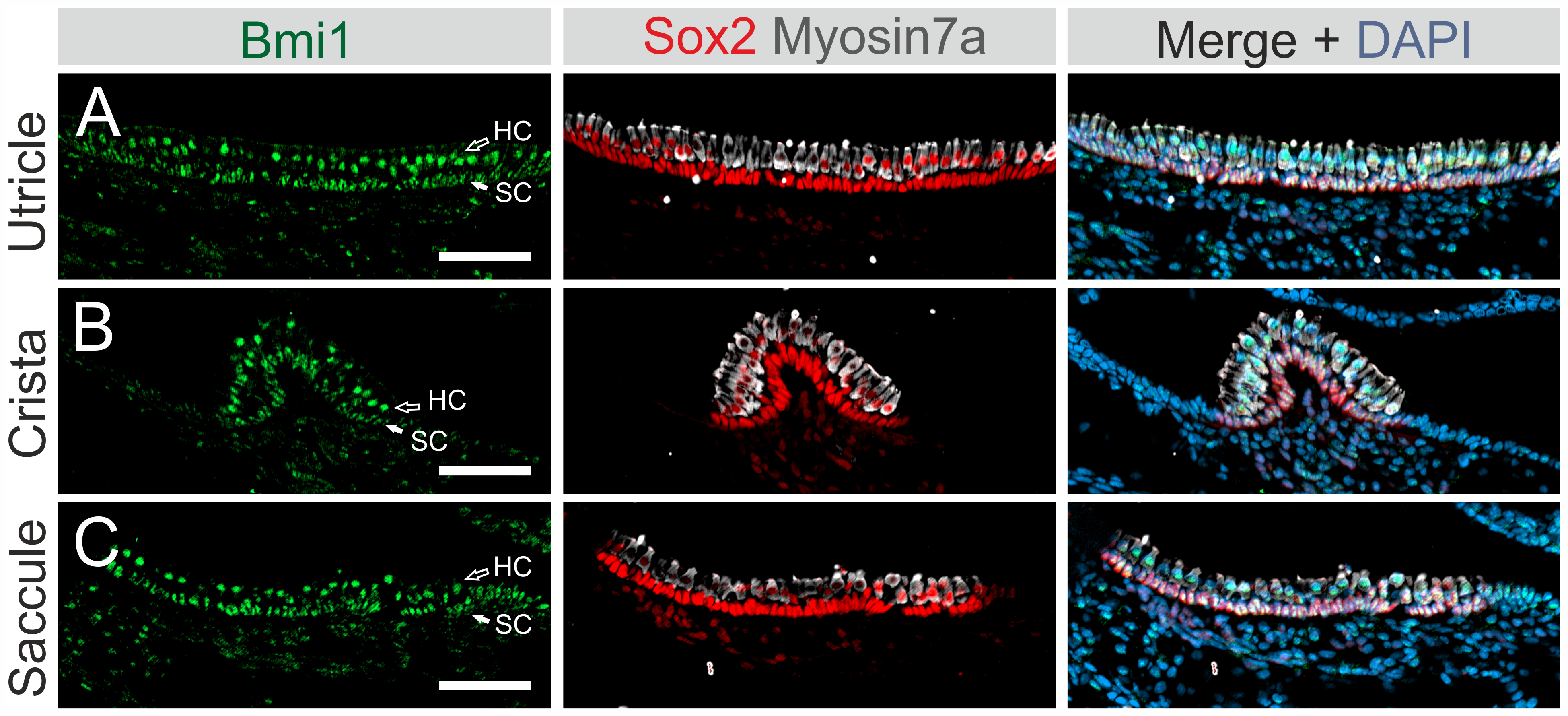

Supplement: S1 Fig — (A-C) Sections of the vestibular apparatus of WT mice at p0, stained for Bmi1 (green) and co-labeled for Sox2 (red) and Myosin7a (white). Nuclei were labeled with DAPI. Bmi1 expression was detected in hair and supporting cells of the utricle (A), crista ampullaris (B) and saccule (C). In all three epithelia, Sox2 expression was observed in supporting cells and a subset of hair cells. Supporting cells are indicated by solid white arrows, while hair cells are indicated by hollow white arrows. SC: supporting cells, HC: hair cells. Scale: 50 μm. (TIF) [file pone.0164579.s001.tif]

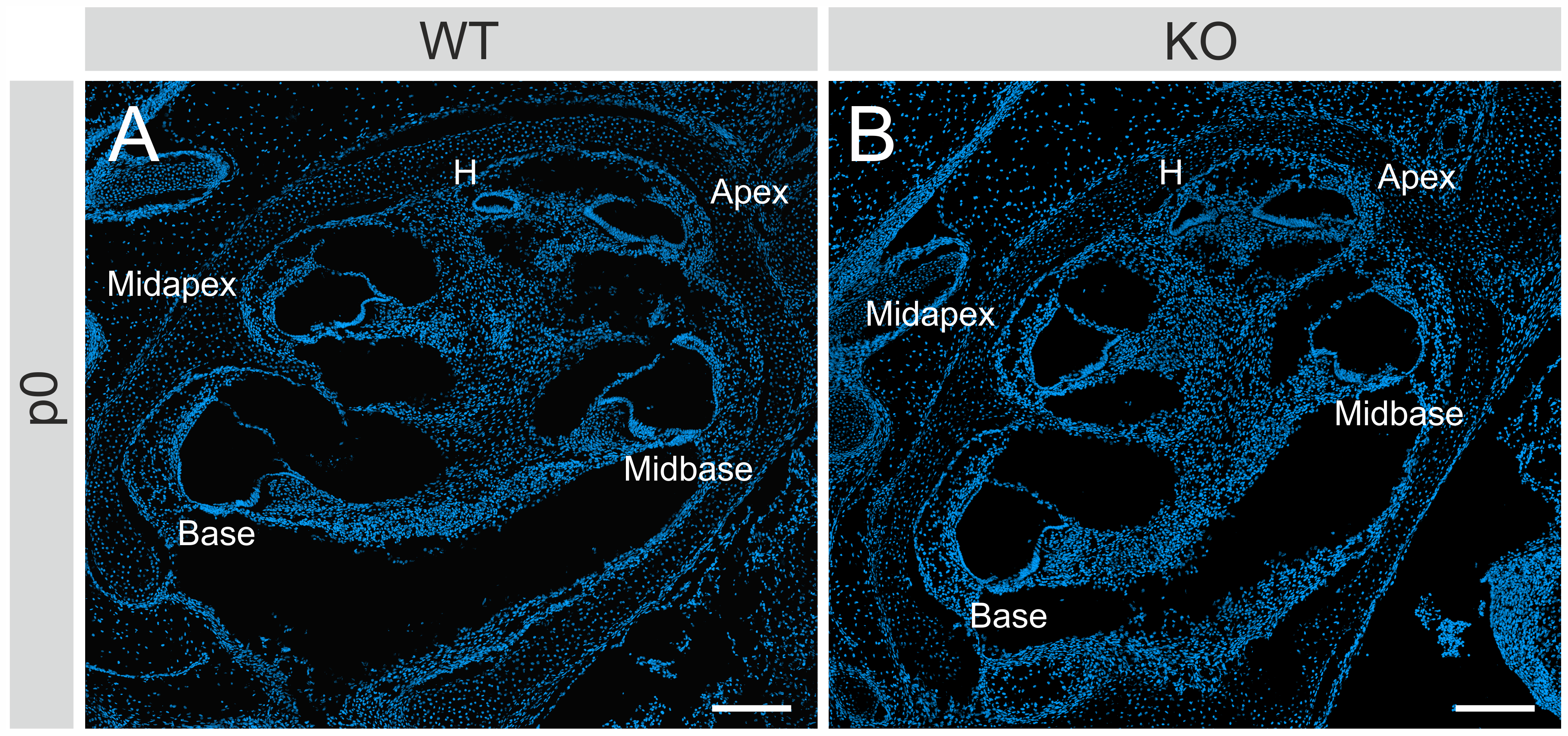

Supplement: S2 Fig — (A and B) Mid-modiolar sections of the cochleae of Bmi1 WT (A) and KO (B) mice at p0, counterstained with DAPI (blue). The KO cochlea displays the normal 4–5 cochlear half-turns. All of the turns appear normally formed, with similar morphology to the WT cochlea. Scale: 200 μm. (TIF) [file pone.0164579.s002.tif]

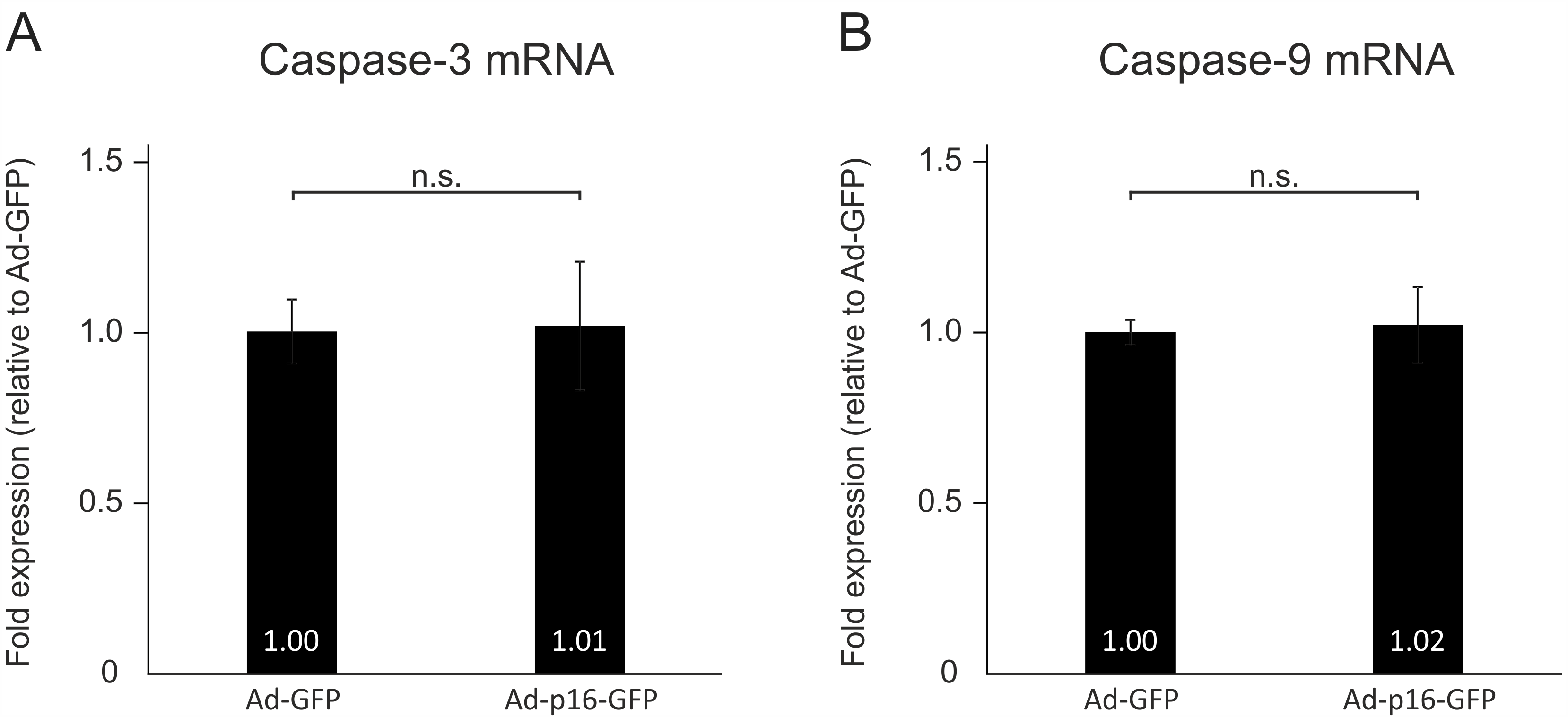

Supplement: S3 Fig — (A and B) Quantitative analysis of caspase-3 and caspase-9 mRNA levels in organ of Corti-derived spheres, which were incubated with either of two viral vectors: i) Ad-GFP to induce the expression of GFP, or ii) Ad-p16-GFP to induce the expression of both GFP and p16ink4a. No significant differences were detected in the levels of caspase-3 (A) or caspase-9 mRNA (B) between the spheres incubated with Ad-GFP and those incubated with Ad-p16-GFP for 5 days in vitro (n = 2 independent samples, measured in triplicate, for both groups, Student’s t-test, p>0.05). n.s.: not significant. (TIF) [file pone.0164579.s003.tif]
