# Supplementary material for: Bmi1 Loss in the Organ of Corti Results in p16ink4a Upregulation and Reduced Cell Proliferation of Otic Progenitors In Vitro
Source: PLoS One. 2016 Oct 18;11(10):e0164579. doi: 10.1371/journal.pone.0164579 (PMC5068820; doi:10.1371/journal.pone.0164579)
Supplement: S1 Table — (DOCX) [file pone.0164579.s004.docx]

**S1 Table: List of antibodies and fluorophores used in this study**

| **Product** | **Host** | **Company** | **Dilution** | **No.** |
| --- | --- | --- | --- | --- |
| Bmi1 | Mouse | Abcam | 1:200 | ab14389 |
| GFP | Rabbit | Invitrogen | 1:400 | A-11122 |
| Myosin7a | Rabbit | Axxora | 1:500 | PTS-25-6790 |
| Myosin7a | Mouse | Developmental Studies Hybridoma Bank | 1:200 | MYO7A138-1 |
| Sox2 | Goat | Santa Cruz Biotechnology | 1:100 | sc-17320 |
| NeuN | Rabbit | Abcam | 1:500 | ab177487 |
| NeuN | Mouse | Merck-Millipore | 1:100 | MAB377 |
| Sox10 | Goat | Santa Cruz Biotechnology | 1:100 | sc-17342 |
| Ki67 | Rabbit | Abcam | 1:200 | ab16667 |
| pHH3 | Rat | Sigma-Aldrich | 1:200 | H9908 |
| EdU "Click-iT® Alexa Fluor® 594“ |  | Thermo Fisher Scientific |  | C10339 |
| Phalloidin Alexa Fluor® 568 |  | Thermo Fisher Scientific | 1:400 | A-12380 |
| DAPI |  | Thermo Fisher Scientific | 0.3 µM | D1306 |
| Anti-mouse Alexa Fluor® 488 | Donkey | Thermo Fisher Scientific | 1:400 | A-21202 |
| Anti-goat Alexa Fluor® 594 | Donkey | Thermo Fisher Scientific | 1:400 | A-11058 |
| Anti-rabbit Alexa Fluor® 647 | Donkey | Thermo Fisher Scientific | 1:400 | A-31573 |
| Anti-rabbit Alexa Fluor® 488 | Donkey | Thermo Fisher Scientific | 1:400 | A-21206 |
| Anti-mouse Alexa Fluor® 647 | Donkey | Thermo Fisher Scientific | 1:400 | A-31571 |
| Anti-rabbit Alexa Fluor® 546 | Donkey | Thermo Fisher Scientific | 1:400 | A-10040 |
| Anti-rat Alexa Fluor® 594 | Donkey | Thermo Fisher Scientific | 1:400 | A-21209 |
